# Effectiveness of an emotion focused cognitive-behavioral therapy (ECBT) program for externalizing disorders in children and adolescents : clinical profile

**DOI:** 10.1192/j.eurpsy.2022.1120

**Published:** 2022-09-01

**Authors:** N. Arfaoui, M. Hajri, Z. Abbes, S. Halayem, A. Bouden

**Affiliations:** 1 Razi Hospital, Child And Adolescent Psychiatry, Tunis, Tunisia; 2 Razi Hospital, Child And Adolescent Psychiatry, Manouba, Tunisia; 3 Razi Hospital, Child And Adolescent Psychiatry, manouba, Tunisia; 4 Razi hospital, Child And Adolescent Psychiatry, manouba, Tunisia

**Keywords:** clinical, externalizing behaviors, emotion regulation

## Abstract

**Introduction:**

Externalizing disorders involve undercontrolled, impulsive, or aggressive behavior. Included in this category are Conduct Disorder, Oppositional Defiant Disorder, and Attention deficit hyperactivity. Difficulties with emotion regulation are a core feature of externalizing disorders in children and adolescents. Yet, no studies to date have compared the relative efficacy of an ECBT program in this population.

**Objectives:**

to investigate the effectiveness of an ECBT inspired program in children and adolescents with Attention Deficit Hyperactivity Disorder (ADHD), Conduct Disorder (CD) and Oppositional Defiant Disorder (ODD)

**Methods:**

We conducted an experimental study with a pretest posttest design and a control group. 50 subjects with either ADHD, ODD or CD were selected and assigned to the experimental and control group. 25 patients ages 9–18 (13 boys, 12 girls) were enrolled in the ECBT-inspired program with 19 completing treatment. Comparison of pre- and post-test results for each sub-group was performed using the Wilcoxon test.

**Results:**

showed that youths in the ADHD and ODD groups demonstrated a significant reduction in externalizing behavior problems measured by the Child Behavior Checklist (CBCL). In terms of emotional regulation, only the group of patients with ODD showed a significant improvement in the cognitive reappraisal subscale of the emotional regulation questionnaire(ERQ- CA ). Only in the ODD group, significant improvement was found in the identification and external oriented thniking subscale scores of the alexithymia questionnaire for children (AQC).

**Conclusions:**

Such comparisons are necessary to determine the clinical profile of patients who might most benefit from such an intervention.

**Disclosure:**

No significant relationships.

